# A proteomic analysis of an *in vitro* knock-out of miR-200c

**DOI:** 10.1038/s41598-018-25240-y

**Published:** 2018-05-02

**Authors:** Bojan Ljepoja, Jonathan García-Roman, Ann-Katrin Sommer, Thomas Fröhlich, Georg J. Arnold, Ernst Wagner, Andreas Roidl

**Affiliations:** 1 Pharmaceutical Biotechnology, Department of Pharmacy, Ludwig-Maximilians-Universität München, Munich, Germany; 2 Laboratory for Functional Genome Analysis (LAFUGA), Gene Center, Ludwig-Maximilians-Universität München, Munich, Germany

## Abstract

Loss of miR-200c is correlated to advanced cancer-subtypes due to increased EMT and decreased treatment efficacy by chemotherapeutics. As miRNAs regulate a multitude of targets, the analysis of differentially expressed proteins upon a genomic knock-out (KO) is of interest. In this study, we generated a TALENs KO of miR-200c in MCF7 breast cancer cells, excluded its compensation by family-members and evaluated the impact on the proteome by analyzing three individual KO-clones. We identified 26 key proteins and a variety of enrichments in metabolic and cytoskeletal pathways. In six of these targets (AGR2, FLNA/B, ALDH7A1, SCIN, GSTM3) the differential expression was additionally detected at mRNA level. Together, these alterations in protein abundance accounted for the observed biological phenotypes, i.e. increased migration and chemoresistance and altered metabolism, found in the miR-200c-KO clones. These findings provide novel insights into miR-200c and pave the way for further studies.

## Introduction

MicroRNAs (miRNAs) are short non-coding RNAs which are known to regulate protein expression at the translational level via base pairing to mRNA or by induction of mRNA decay^1,2^. Since their discovery, miRNAs have had a tremendous impact on our understanding of physiology and pathophysiology, leading to ever increasing efforts to discover miRNA genes, their function and targets^3,4^. MiRNAs are important for a broad spectrum of biological processes, such as embryonic development, immune differentiation, metabolism and cardiac function^5–8^. On the other hand, their aberrant expression is involved in a vast number of diseases, such as diabetes and cancer^5,9,10^. Therefore, miRNAs are promising tools as biomarkers or therapeutic agents^11^.

An important group of miRNAs in the context of cancer research is the miR-200 family, consisting of miR-200a, miR-200b, miR-200c, miR-141 and miR-429. Many family members are known to play a role in a large variety of biological processes like Epithelial to Mesenchymal Transition (EMT), cell invasion, proliferation, metastasis, apoptosis, autophagy, and therapy resistance in several cancer types^1,12–16^. MiR-200c is the most prominent member in tumorigenesis, as its role in several hallmarks of cancer, such as EMT, chemoresistance, migration and stemness^1,17^, has already been described. Although the involvement of miR-200c in these processes was demonstrated, many underlying mechanisms and players remain unknown^1^, especially in controversially discussed processes like chemoresistance or proliferation^1,18–21^.

In our previous work, we were able to show the involvement of miR-200c in sensitizing breast cancer cells to doxorubicin, via regulating BMI1 and TRKB^19^ as well as the direct interaction of miR-200c with the mRNA of the prominent oncogene KRAS^20^.

The vast majority of the studies analyzing the biology of miR-200c utilizes short-term inhibition approaches making use of LNAs or antagomirs, but omitting the impact of miR-200c depletion in the long-term^19,22,23^. The latter reflects the loss of miR-200c expression in a tumor, as is frequently observed in the clinics^24,25^. Thus, analyzing the knock-out (KO) of miR-200c leads to novel insights into miR-200c’s role in advanced breast cancer.

With current genome editing tools like TALENs (Transcription Activator-Like Effector Nucleases) and CRISPR-Cas9 (Clustered Regularly Interspaced Short Palindromic Repeats)^26,27^ a revolution in many fields of gene research was initiated. While both tools have different properties and demand different strategies, they also equally harbor high potential for research of non-coding genes, like miRNAs. TALEN are fusion proteins that induce a double strand break in the DNA, but have to be designed as pair, specifically targeting the desired genomic site. The nuclease-activity will cause a double strand break (DSB) which can be repaired on the one hand by error prone Non-Homologous End Joining (NHEJR), in most cases leading to an indel formation and thus to the knock-out of the gene. On the other hand Homologous Recombination (HR) results in successful repair of the DSB^27,28^.

CRISPR-Cas9 is based on the nuclease-activity of Cas9, but the targeting is initiated by short-guiding RNAs (sgRNA) and is limited to genomic sites with a protospacer adjacent motif (PAM). CRISPR approaches usually lead to a double strand break and only one sgRNA needs to be designed^26,29,30^.

In this study, we utilized TALENs for a genetic KO of miR-200c due to its flexibility to target any genomic sequence. This approach allowed us to develop a long-term *in vitro* model of miR-200c depletion (KO) in MCF7 breast cancer cells. With a subsequent proteomic analysis, we were able to gain novel insights into changes in the proteome, i.e. differentially expressed proteins, resulting from the absence of only 22 non-coding basepairs of the miR-200c.

## Results

### A miR-200c knock-out - strategy and validation

**Figure 1:**
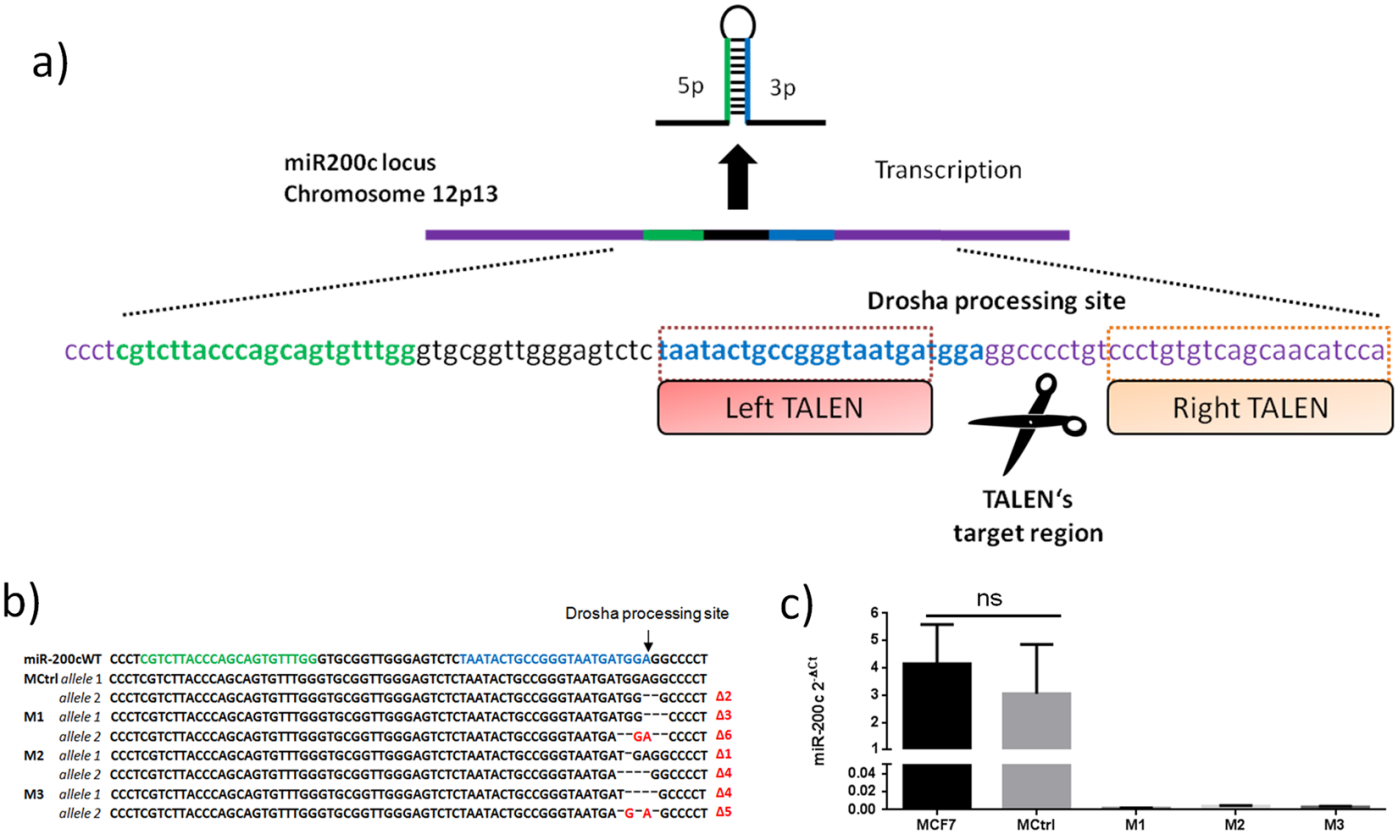
miR-200c genetic TALEN target sequences and knock-out confirmation. (**a**) The miR-200c gene is located at chromosome 12p13. TALENs were designed to target the miR-200c 3p drosha processing site. (**b**) Genomic DNA was extracted from MCtrl and M1, M2 and M3 clones, afterwards the miR-200c gene was amplified by PCR to perform sequencing of the miR-200c loci; MCtrl shows a heterozygous mutation while M1, M2 and M3 show various indels in the proximity of the miR-200c 3p drosha processing site on both alleles. (**c**) MCF7, MCtrl, M1, M2 and M3 miR-200c expression levels were analyzed by quantitative RT-PCR. Expression of miRNAs is shown as mean of three independent experiments ± SD. ns: no statistical difference, p > 0.05, one-way ANOVA post hoc Bonferroni.

To generate the miR-200c KO, we chose to genetically disrupt the drosha processing site. Generally two options for genomic editing were available – CRISPR/Cas9 and TALENs. While a PAM-sequence was present in the drosha processing site, suitable sgRNAs were designed with the CRISPR-design tool^31^ but resulted in 60–75 off-targets, amongst them 6–9 in coding regions. Utilizing a Cas9-Nickase would result in lower off-targets, but it was not possible to design a pair inducing a site-specific mutation within the limited number of base-pairs of the drosha-site. Therefore, we chose a pair of TALENs to disrupt miR-200c 3p gene expression by targeting the flanking regions of the drosha processing site as described previously^32^. Eventually we sought to induce a double strand break in the vicinity of the drosha processing site (Fig. 1a). MCF7 cells were chosen as model for an epithelial breast cancer cell line with high miR-200c expression^19^. A single cell dilution was performed and clones were selected to sequence indel formation at the genomic locus of the miR-200c drosha site. Three of the monoclonal cell lines, namely M1, M2 and M3 showed deletions in both alleles of the miR-200c gene which were located in vicinity of the drosha processing site, i.e. homozygous KO of miR-200c. One clone (MCtrl) showed a heterozygous mutation (Fig. 1b).

A qPCR-measurement of miR-200c expression of M1, M2 and M3, confirmed the knock-out of the miR-200c gene (Fig. 1c). The heterozygous mutations in MCtrl had no significant effect on the miR-200c expression, as levels were comparable to MCF7 wild-type (p > 0.05). Therefore, besides wild-type MCF7, MCtrl was considered as additional control for further analysis.

### Unchanged expression of miR-200 family members

**Figure 2:**
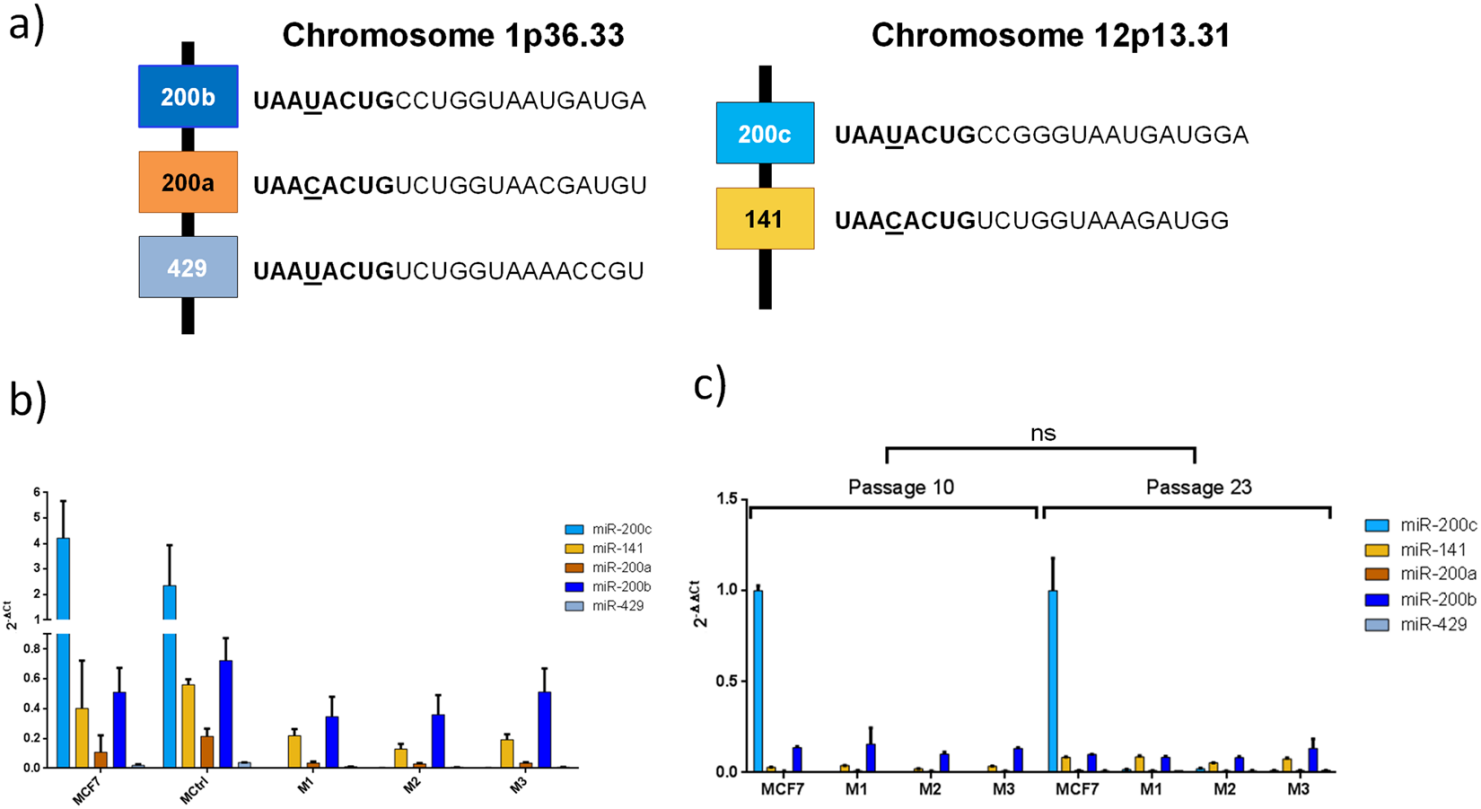
Expression of miR-200 family members among the KO clones. (**a**) The miR-200 family is distributed on two chromosomes; miR-200b, miR-200a and miR-429 are located on chromosome 1p36.33 and miR-200c and miR-141 are located on chromosome 12p13.31. miRs with the same seed region are colored in tones of orange or blue respectively. (**b**) MCF7, MCtrl, M1, M2 and M3 were analyzed for miR-200c, miR-141, miR-200a, miR-200b and miR-429 relative expression levels by quantitative RT-PCR. (**c**) Cells from passage 10 and passage 23 were compared regarding their miR-200c, miR-141, miR-200a, miR-200b and miR-429 relative expression levels via quantitative RT-PCR. Expression of miRNAs is shown as mean ± SD of three independent replicas. ns means no statistical difference, p > 0.05, three way ANOVA post hoc Bonferroni.

To investigate possible compensation effects of the knock out, we analyzed the expression levels of the other miR-200c family members. The genomic loci are comprised of two genomic clusters, one located at chromosome 1p36 including miR-200a, miR-200b and miR-429, and one on chromosome 12p13 containing miR-200c and miR-141^1^. MiR-200c shares the same seed region with miRs 200b and 429 (Fig. 2a).

Subsequently, a qPCR analysis of the expression of all family members was performed.

This data showed that miR-200c is the family member with the highest expression in MCF7. Further analysis revealed that no family member was compensating for the loss of miR-200c by an increase of expression and no general upregulation of all family members was observed (p > 0.05) compared to the control group (MCF7 and MCtrl). Of note, also the expression levels of miR-141 remained similar, i.e. not influenced by the KO of miR-200c, despite the localization in the same polycistronic unit (Fig. 2b).

The knock-out of a miRNA is fundamentally different to its short term inhibition, giving the cells more time to compensate the loss of miR-200c. Therefore, late compensatory mechanisms were ruled out by re-evaluation of the expression of miR-200c family members at a late cell passage number. Compared to earlier passages, the data showed no remarkable changes in the different clones over time (p = 0.896, Fig. 2c). The slight increase of miR-141 is not significant.

### Proteomic analysis of three individual KO clones resulted in 26 novel targets

**Figure 3:**
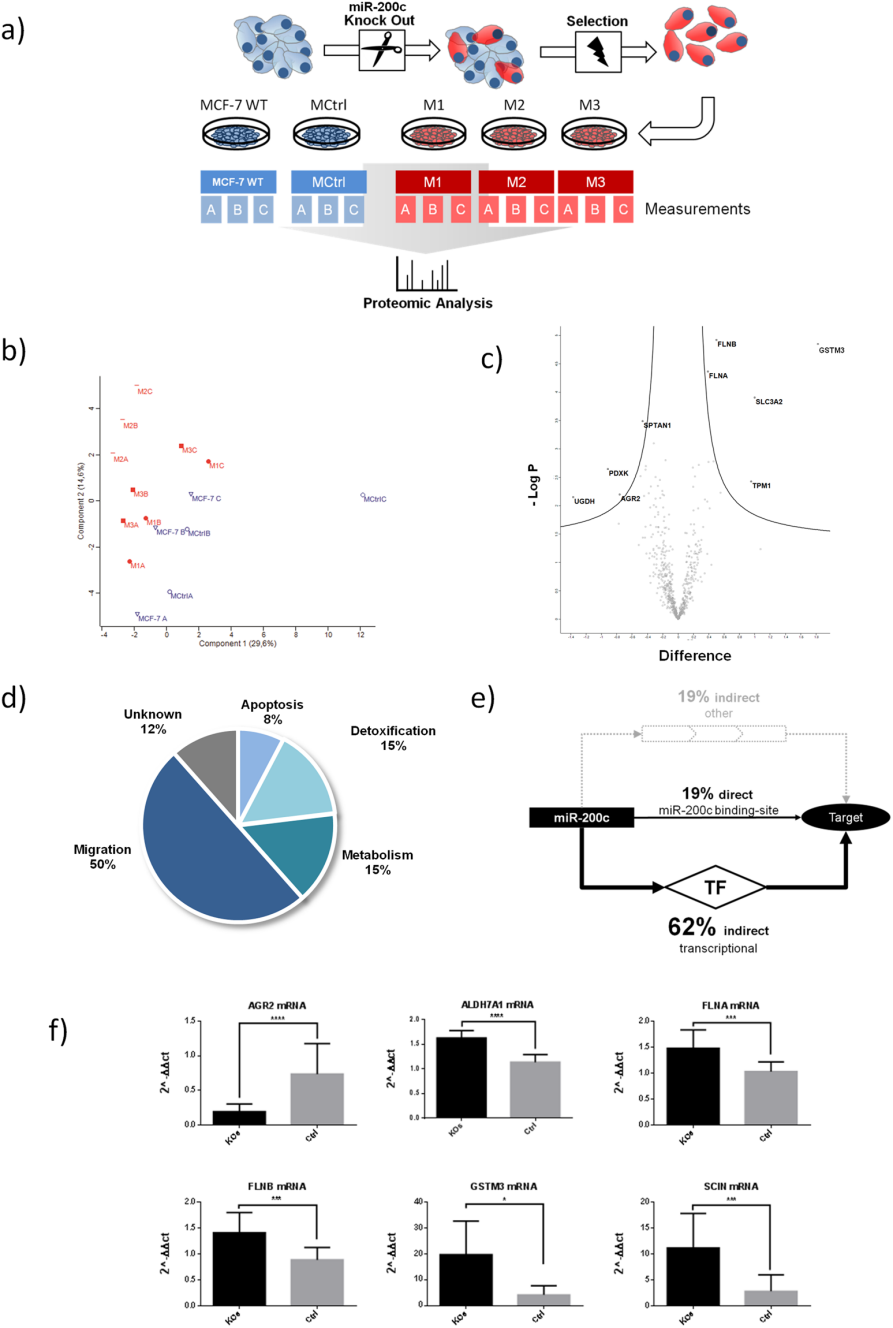
Proteomic analysis of three different KO clones. (**a**) Schematic overview of the experimental set-up. Each clone was measured in independent replicas. (**b**) Principal component analysis of the measurements, KOs are shown in red, Ctrls in blue. (**c**) Volcano plot analysis of grouped controls: (MCF7 WT A/B/C and MCtrl A/B/C) vs. (M1 A/B/C and M2 A/B/C and M3 A/B/C), N = 675 with 250 randomizations, FDR 0.05 and S0 of 0.1. (**d**) Percentage of main functional pathways of targets in Tables 1–3, as derived from the Uniprot-Database. (**e**) Analysis for possible seed-interaction of miR-200c with the targets of Tables 1–3 and further analysis of not-directly regulated targets for binding of transcription factors with predicted miR-200c regulation, see also supporting information Table S3. (**f**) Validation of mRNA expression with grouped statistical analysis (M1 and M2 and M3 vs MCF7 WT and MCtrl, N = 9 (KO)/6 (Ctrl)) for the anterior gradient protein 2 homolog, aldehyde dehydrogenase 7 family member A1, filamin A and B, glutathione S-transferase M3 and adseverin, *p ≤ 0.05 ***p ≤ 0.001 ****p ≤ 0.0001.

To evaluate the effect of the miR-200c KO on a wide range of proteins, a proteomic approach was chosen (Fig. 3a): All clones (M1, M2, M3, MCtrl), as well as wild-type MCF7 cells were harvested in three replicas (A/B/C), and subsequently, proteomic data analysis was performed, resulting in a set of 1736 identified proteins. For the following analysis, we chose to narrow the set down to proteins that were identified in every single measurement.

This filtering resulted in a subset of 675 proteins. With this subset, a principal component analysis (PCA) was performed to investigate the similarity of the clones and replicas. Figure 3b shows a general trend of grouping of the replicas (with exception of MCtrl C) as well as a closer relation between the KO-clones M1, M2 and M3 and the controls MCF7 and MCtrl, respectively. A similar picture is seen in a cluster analysis, as shown in the supporting information S1. For statistical evaluation of differentially expressed proteins a Volcano plot analysis was performed (Fig. 3c), comparing the expression of the KO-clones M1, M2 and M3 (KO) to the controls MCF7 and MCtrl (Ctrl). This analysis revealed nine proteins with significant changes in regulation as shown in Table 1.

**Table 1:** Targets with significant difference between both groups – M1 and M2 and M3 vs MCF7 and MCtrl.

Protein	Gene	p-Value	fold change	Expression	Function
Anterior gradient protein 2 homolog	**AGR2**	6.36 × 10^−3^	0.59	down	Migration
Filamin-A	**FLNA**	4.32 × 10^−5^	1.31	up	Migration
Filamin-B	**FLNB**	1.20 × 10^−5^	1.41	up	Migration
Glutathione S-transferase Mu 3	**GSTM3**	1.43 × 10^−5^	3.54	up	De-Tox
Pyridoxal kinase	**PDXK**	2.27 × 10^−3^	0.53	down	Metabolism
4F2 cell-surface antigen heavy chain	**SLC3A2**	1.25 × 10^−4^	2.00	up	Apoptosis
Spectrin alpha chain, non-erythrocytic 1	**SPTAN1**	3.26 × 10^−4^	0.73	down	Migration
Tropomyosin alpha-1 chain	**TPM1**	3.74 × 10^−3^	1.93	up	Migration
UDP-glucose 6-dehydrogenase	**UGDH**	7.10 × 10^−3^	0.39	down	Migration

To investigate effects on single-clone level, a further T-test with the same parameters was performed, comparing each KO-Clone (M1, M2 or M3) to the grouped controls (e.g. M1 vs. MCtrl and MCF7). The analysis revealed 17 significant hits, as shown in Figure S2 and summarized in Table 2. Here, M2 is pointed out as most diverse from the controls with 14 proteins being differentially expressed, while the two other clones show only statistical difference in one or six proteins for M1 and M3, respectively (supporting Figure S2).

**Table 2:** Targets with significant difference between control and at least one clone: M1 or M2 or M3 vs. MCF7 and MCtrl.

Protein names	Gene	p-Value	fold increase	Expression	Function
Anterior gradient protein 2 homolog	**AGR2**	M2 vs Ctrl	M2 vs Ctrl	down	Migration
		2.82 × 10^−4^	0.397		
Alpha-aminoadipic semialdehyde dehydrogenase	**ALDH7A1**	M2 vs Ctrl	M2 vs Ctrl	up	De-tox
		4.69 × 10^−4^	1.96		
Carbonic anhydrase 2	**CA2**	M2 vs Ctrl	M2 vs Ctrl	down	Unknown
		4.07 × 10^−3^	0.361		
Src substrate cortactin	**CTTN;EMS1**	M2 vs Ctrl	M2 vs Ctrl	down	Migration
		4.67 × 10^−4^	0.743		
Aspartate aminotransferase	**GOT2**	M3 vs Ctrl	M3 vs Ctrl	down	Metabolism
		1.38 × 10^−4^	0.556		
Glutathione S-transferase Mu 3	**GSTM3**	M1 vs Ctrl	M1 vs Ctrl	up	De-tox
		6.03 × 10^−5^	2.48		
		M2 vs Ctrl	M2 vs Ctrl		
		1.50 × 10^−7^	6.17		
		M3 vs Ctrl	M3 vs Ctrl		
		1.21 × 10^−5^	2.91		
Heat shock protein HSP 90-alpha	**HSP90AA1**	M2 vs Ctrl	M2 vs Ctrl	up	Metabolism
		4.64 × 10^−4^	1.39		
D-3-phosphoglycerate dehydrogenase	**PHGDH**	M2 vs Ctrl	M2 vs Ctrl	up	Metabolism
		6.57 × 10^−3^	1.89		
Kynureninase	**KYNU**	M2 vs Ctrl	M2 vs Ctrl	up	Metabolism
		1 × 10^−3^	1.61		
DNA replication licensing factor MCM4	**MCM4**	M2 vs Ctrl	M2 vs Ctrl	down	De-tox
		2.12 × 10^−4^	0.761		
Ras-related protein Rab-14	**RAB14**	M2 vs Ctrl	M2 vs Ctrl	down	Migration
		1.23 × 10^−5^	0.595		
SH3 domain-binding glutamic acid-rich-like protein	**SH3BGRL**	M3 vs Ctrl	M3 vs Ctrl	up	Migration
		4.01 × 10^−5^	1.85		
4F2 cell-surface antigen heavy chain	**SLC3A2**	M2 vs Ctrl	M2 vs Ctrl	up	Apoptosis
		7.09 × 10^−6^	2.91		
		M3 vs Ctrl	M3 vs Ctrl		
		6.42 × 10^−4^	1.71		
Triosephosphate isomerase	**TPI1**	M3 vs Ct	M3 vs Ctrl	down	Metabolism
		7.95 × 10^−5^	0.69		
Tropomyosin alpha-1 chain	**TPM1**	M2 vs Ctrl	M2 vs Ctrl	up	Migration
		4.76 × 10^−3^	2.71		
UDP-glucose 6-dehydrogenase	**UGDH**	M2 vs Ctrl	M2 vs Ctrl	down	Migration
		3.67 × 10^−6^	0.155		
		M3 vs Ctrl	M3 vs Ctrl		
		3.28 × 10-3	0.533		
Tryptophan-tRNA ligase	**WARS**	M2 vs Ctrl	M2 vs Ctrl	up	De-tox
		1.20 × 10^−3^	1.58		

 To further analyze targets which may have had changes in expression in response to the miR-200c KO but have not been detected in the previous analysis, we searched for proteins that were not detected in the KO-group, but were found in the control-group (found at least 3 times in Ctrl, not at all in the KO). These proteins were termed “OFF”. Vice versa, “ON” proteins display no expression in the control-group, but are expressed in the KO-group (at least 5 times in KO, but not in Ctrl). Table 3 lists the three targets gaining expression (ON) and the two proteins losing expression (OFF) after the KO.

**Table 3:** Targets detected in just one of the groups: M1 and M2 and M3 OR MCF7 and MCtrl.

Protein	Gene	Expression	Function
N-acetylserotonin O-methyltransferase-like protein	**ASMTL**	ON	Unknown
Serine/threonine-protein phosphatase PP1-gamma catalytic subunit	**PPP1CC**	OFF	Unknown
Apoptosis-associated speck-like protein containing a CARD	**PYCARD**	OFF	Apoptosis
Regulator of microtubule dynamics protein 1	**RMDN1**	ON	Migration
Adseverin	**SCIN**	ON	Migration

The altogether 26 targets shown in Tables 1, 2 and 3 were grouped according to their main function as stated by the Uniprot-Database^33^. As shown in Fig. 3d, more than half of the proteins are found in migratory processes and metabolism (45% and 17% respectively), while other functions are detoxification (10%) and apoptosis (11%), with remaining 17% of proteins, with no known function.

### Analysis of the targets for miR-200c regulation

To evaluate whether the targets are directly regulated by miR-200c, the genes were analyzed for binding sites with the TargetScan database. One fifth of the proteins harbor a potential targeting site (8mer or 7mer-m8/A1 seed-region match) in their 3′UTR. For the remaining 21 genes, a possible promotor binding site of miR-200c regulated transcription factors was investigated. This analysis revealed that 62% of the genes without binding site may be indirectly regulated by miR-200c: The promotor region of these genes contains at least one putative binding site for a transcription factor which is potentially regulated by miR-200c (Fig. 3e and supporting information Table S3).

Further, we measured whether the altered protein-levels resulted from changes of the mRNA levels. Therefore, we compared mRNA levels of the single clones to their proteomic data (see supporting information S3). For six targets alterations in protein abundance were reflected at the mRNA level (Fig. 3f).

AGR2, the anterior gradient protein 2 homolog, was found to be statistically significant differentially expressed in the proteomic analysis in Table 1. The mRNA expression correlates with the protein expression from the proteomic approach and the grouped analysis, i.e. M1 and M2 and M3 vs MCF7 WT and MCtrl, showed an almost four-fold increase with a highly significant difference between KO and Ctrls respectively (p ≤ 0.0001). Furthermore, aldehyde dehydrogenase 7 family member A1, ALDH7A1’s protein expression changed significantly in a part of the clones, but on mRNA it shows highly significant (p ≤ 0.0001) increased expression of 43%. Additionally, Filamins FLNA and FLNB were found to be significantly changed on protein level (Table 1). Again, on mRNA level both filamins show a significant (p = 0.0004 / p = 0.0003) increase of 44% and 59% in FLNA and FLNB respectively. Glutathione S-transferase Mu3 shows an increase in the mRNA expression compared to the controls (p = 0.138). SCIN, adseverin protein from the “ON” target list (Table 3), also showed a four-fold increase in the KO compared to the controls (p = 0.0004). Taken together in these six cases, the mRNA-measurements indicate a regulation of these targets on mRNA level.

### The KO of mir-200c results in changes in cellular processes and pathways

**Figure 4:**
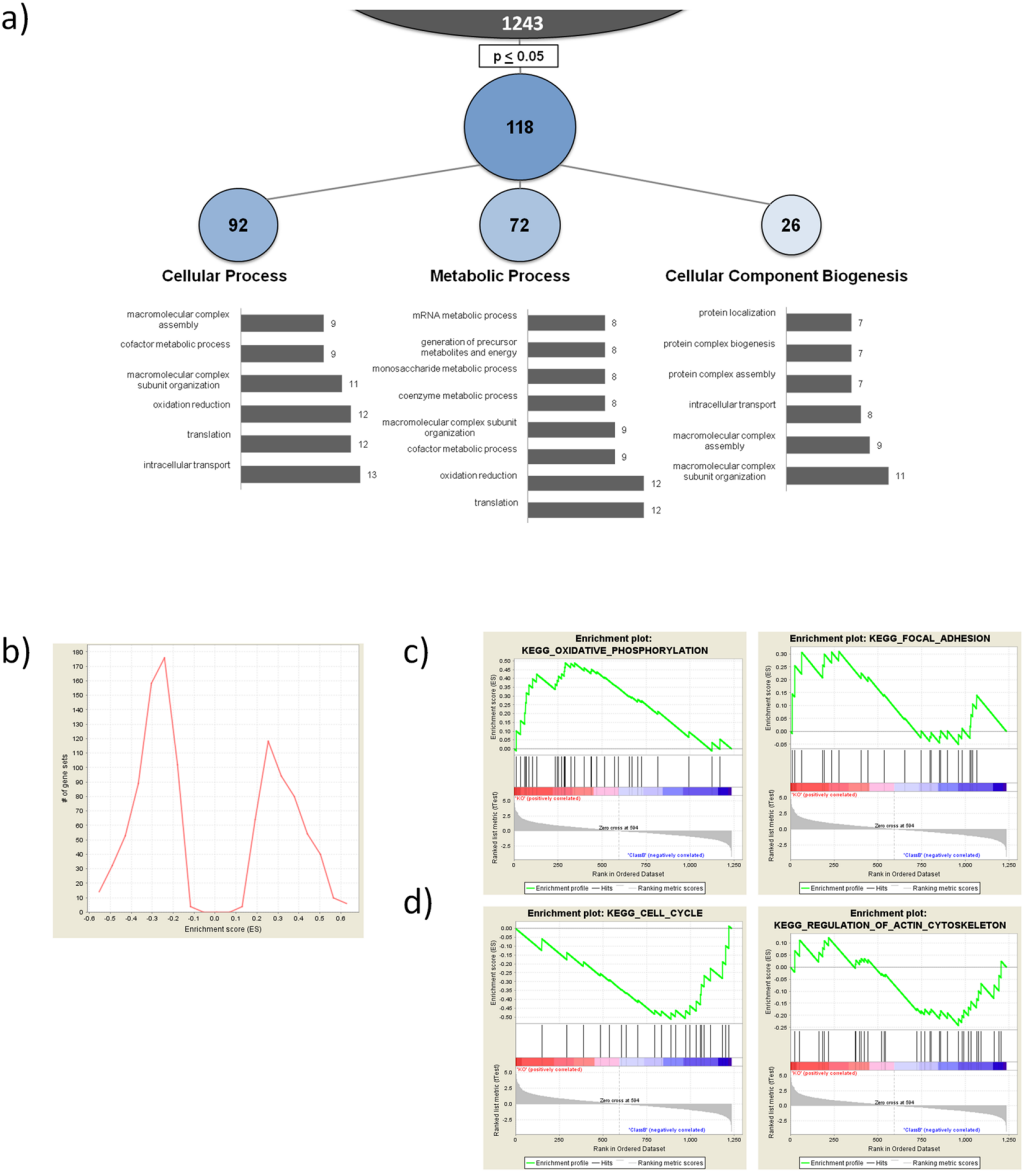
Bioinformatic analysis of the proteomic dataset. (**a**) Targets for DAVID 6.7 analysis with the GOterm BP database were chosen from the whole dataset for every protein with p ≤ 0.05 after student’s t-test KO vs Ctrl (**b**) Distribution of ES Scores in a GSEA of KO vs Ctrl with Gene Ontology (c5.all.v5.2) and KEGG (c2.cp.kegg.v5.2) reference database (**c**) GSEA Enrichment-Plot analysis of the whole dataset shows two exemplary KEGG pathways. Oxidative phosphorylation and focal adhesion showing overexpression while (**d**) cell cycle and regulation of actin cytoskeleton are being down regulated.

For a broader analysis of changes in biological processes and pathways, the original dataset was filtered for proteins that appeared at least three times in at least one group (i.e. three times in KO or Ctrl). This resulted in a new subset of 1243 proteins. Missing values were replaced by the imputation algorithm of Perseus. After a two tailed t-test comparing KO to Ctrl, all proteins with p ≤ 0.05 were analyzed with the DAVID functional gene annotation tool^34,35^ with the GoTerm BP (Biological Processes) database (N = 118, Fig. 4a).

The majority of functional annotations was categorized to BP1 cellular process (92), metabolic processes (72) and cellular component biogenesis (26). A detailed view on the processes is showing the top most frequent sub-classifications according to the number of attributed genes in GoTerm BP FAT. Prominent processes involve intracellular transport, translation and oxidation reduction as well as macromolecular complex assembly and subunit organization. These findings indicate a broad influence of miR-200c on essential processes.

Moreover, a Gene-Set Enrichment Analysis of the whole dataset after imputation (N = 1243) was performed against GO and KEGG databases. The global Enrichment Score (ES) histogram revealed that miR-200c knock-out resulted mainly in the inactivation of pathways, as shown by an accumulation of negative ES (Fig. 4b). For depicting exemplary pathways, we chose KEGG pathway annotations. Enriched pathways (supporting table S1) contain mainly metabolic processes like oxidative phosphorylation, citrate cycle and glycolysis, or cytoskeletal organsiation as shown in changes in focal adhesion (Fig. 4c). Negative pathway enrichment was observed in adherens junctions and tight junctions, regulation of actin skeleton as well as other metabolic pathways like purine metabolism and decrease in the cell cycle (Fig. 4d and supporting Table S1). Heatmap analysis of the GSEA (supporting Figures S4 and S5) show a high occurrence of targets of Tables 1 and 2 in all of these pathways.

Taken together, the GSEA findings indicate an increase in metabolic pathways, which also may increase de-toxification in the cells as well as numerous de-regulations in cell-cell contacts and cytoskeletal organisation, which may lead to increased metastatic potential.

### Biological assays reveal the impact of the miR-200c KO on EMT, chemoresistance and metabolism

**Figure 5:**
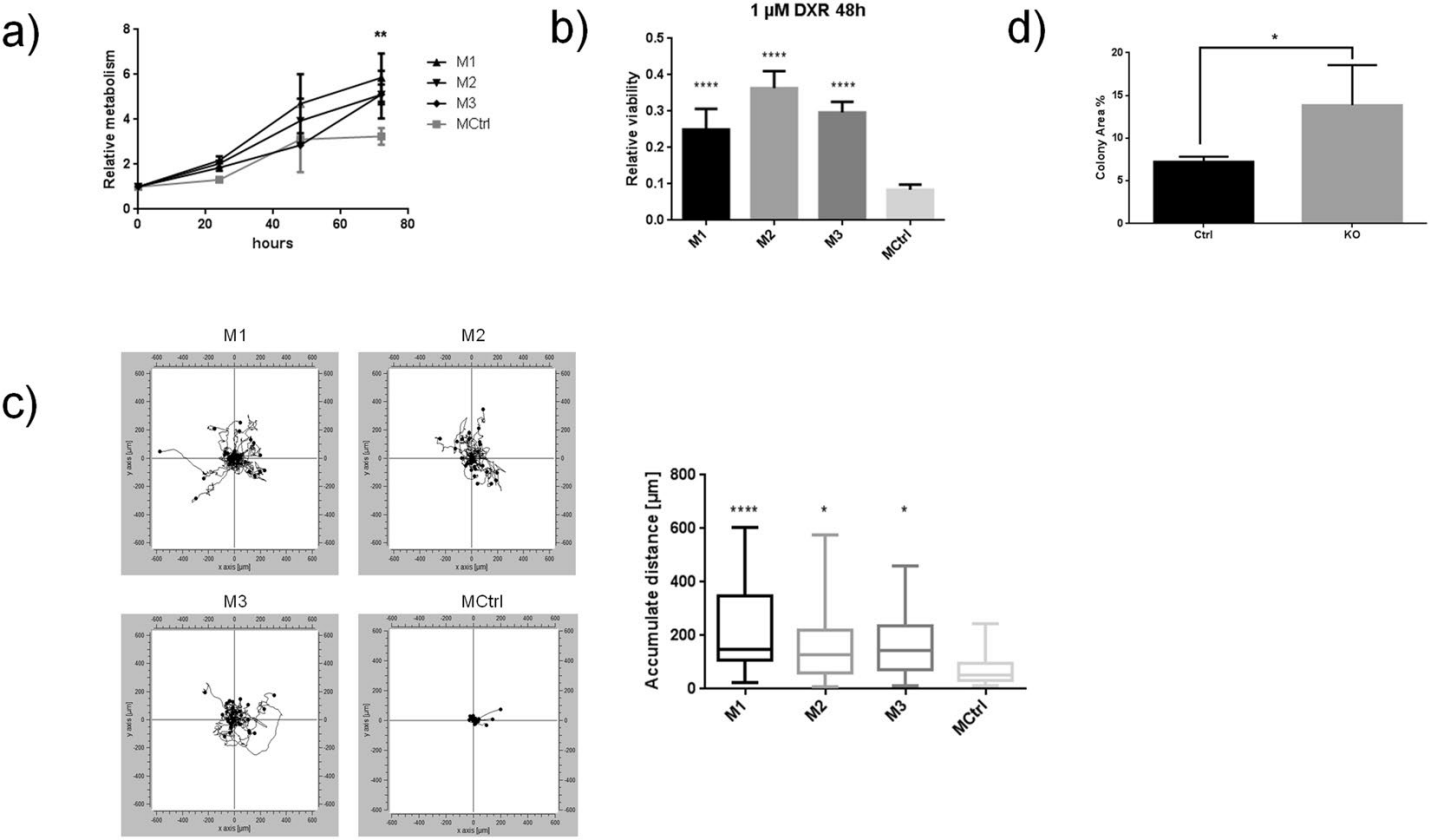
Biological data to validate the predicted phenotype. (**a**) Analysis of the relative increase of metabolic activity via MTT-Assay, normalized to each starting point, ***p ≤ 0.001 for M1 and **p ≤ 0.01 for M2 and M3 compared to MCtrl, N = 4, two-way ANOVA with Dunnett’s multiple comparison. (**b**) Treatment with 1 µM doxorubicine for 48 h and analysis of viability as by CTG assay, N = 6, ****p ≤ 0.0001 compared to Ctrl, two-way ANOVA with Bonferroni’s multiple comparison). (**c**) Analysis of colony forming abilities via the clonogenic assay shows a significantly higher colony area in the KOs after seven days of incubation, student’s t-test, p ≤ 0.05, N = 3 or 9, images in supporting Figure S7. (**d**) Single cell tracking measurement for evaluation of the migratory potential, displayed as accumulative distance after 20 h, N = 30, ****p ≤ 0.0001, *p ≤ 0.05 compared to MCtrl, one-way ANOVA with Dunnett’s multiple comparison after outlier test, velocity displayed in S6.

To confirm the biological relevance of the data, different *in vitro* assays were performed utilizing the clonal cell lines (KOs and MCtrl). The metabolic activity was assessed by measuring NAPD(H)-turnover via MTT assay over the course of 72 h. All clones showed a significantly higher turnover (***p ≤ 0.001 for M1 and **p ≤ 0.01 for M2 and M3), either due to increased metabolic activity or higher proliferation (Fig. 5a). The change of resistance to chemotherapeutics was analyzed by treating the cells with doxorubicin (DXR) analyzing relative viability via the Celltiter-Glo assay (Fig. 5b). The strongest effect was observed in M2, which was almost 4-times higher than MCtrl. Still, also all other clones show a highly significantly increased viability and therefore higher resistance to chemotherapeutics (p ≤ 0.0001).

**Figure 6:**
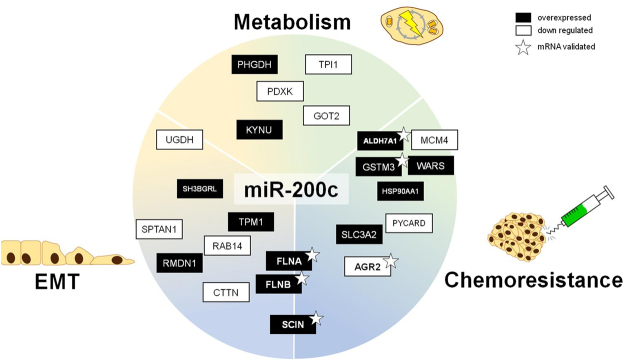
Summary of important pathways and biological phenotypes, with targets from Tables 1–3 matched to their known functions.

Previously described de-regulations in cell-cell contacts and cytoskeleton were analyzed by investigation of colony-formation abilities as well as of the migratory potential. A significant increased colony area (p ≤ 0.05) after seven days was observed in the KO cells (Fig. 5c and supplemental Fig. S7). The live imaging experiment with single cell tracking (20 h, N = 30), as shown in Fig. 5d, indicates that the KO cells show a tendency of migrating further and faster than the Ctrl, with the differences between M2 and M3 to Ctrl being statistically significant (p ≤ 0.05) and M1 to Ctrl being highly significant (p ≤ 0.0001) (additional information in supporting graph S6). While these results indicate EMT, well-known mechanisms, like activation of ZEB1/2 or Vimentin were not detected and E-cadherin levels were not changed (supplemental Figure S8). Taken together, our results show that miR-200c plays a crucial role in cancer progression, by modulating the protein expression leading to a change of fundamental physiological properties, i.e. increasing metabolism and proliferation, the induction of EMT and enabling cell migration as well as increasing chemoresistance (Fig. 6).

## Discussion

Recent publications on the role of miR-200c in cancer progression and metastasis^14,20^ point towards a multilayered and complex interplay^36^, also involving other numerous pathways like angiogenesis and therapy resistance^12,37^. These facts emphasize the need to utilize more comprehensive tools like genomics and proteomics, as key to generate novel insights. With the genomic knock-out, followed by proteome analysis, we chose two state-of-the-art techniques to investigate new modes of action of miR-200c.

While knock-outs of protein coding genes are quite common, the genetic disruption of non-coding regulatory RNAs is still rarely reported. CRISPR/Cas9 is probably the most frequently utilized genome-editing technology at this time, nevertheless this tool’s main disadvantage lies in the tendency to off-target cleavage^26,29^. Also, the need of a PAM-sequence may impede certain knock-out strategies, especially when site-specific mutations are necessary. While different modifications were performed to enhance Cas9’s specificity, like the conversion to the Cas9n nickase-mutant or rational design of the nuclease^31,38^, TALENs offer high specificity from the beginning, as was also demonstrated by successful use in a first human patient^39^. Therefore, in our experiment we chose to utilize TALENs, allowing us to specifically target the miRNA’s drosha processing site^27,32^. The genetic disruption in the drosha site minimizes the risk of inducing a mutation in the seed region, which could lead to the generation of a new, artificial miRNA with unpredictable off-targets. Kim *et al*. provided the pre-designed TALENs-plasmids, and previously showed that a mutations of the drosha processing site leads to a decrease in miR-expression. Further, the group was able to verify the KO-strategy for miR-200c by demonstrating effects of the miR-200c KO in the Her2-positive SKBR3 cell line, like an increase of the miRNA’s seed-targets via a motif enrichment analysis and decreased proliferation^32^.

In our work, we were able to generate mutations in both alleles of miR-200c in three independent clones, namely M1, M2 and M3, as well as one clone with a heterozygous mutation, i.e. MCtrl. As miR-200c family members share most of their sequence and were reported to have similar functions^12,13^, and as the knock-out of a gene can induce compensation effects^40^, it was necessary to analyze the expression of the family members after the knock-out of miR-200c. The measurements emphasize the general importance of miR-200c among its family members in this cell line, as the levels are about 20-fold higher than the average miR-200c-family members. No significant transcriptional compensation of any other family member was observed. In MCtrl the compensation of the loss of one-allele could be based on a higher transcription rate of the polycistronic unit, which would result in higher levels of miR-141. However, the observed increase in miR-141 expression is not significantly higher and does not correlate with the increase needed for the compensation of the loss of one allele of miR-200c needed. These findings together suggest a different compensatory mechanism in MCtrl, like inhibited degradation or changes in the miRNA processing of miRNA-200c. Still, it is not clear whether the basal expression levels of miR-200b and miR-429, which share the same seed region as miR-200c, could suffice for the regulation of certain targets and pathways.

Only few miRNA-knock-outs, especially with TALENs, were described before^41,42^, but the subsequent target analysis has been mainly based on genomic approaches. The protein expression profile analysis therefore may reveal important novel information about the regulation network of miRNA-200c. In the proteomic approach, three knock-out clones were analyzed and compared to both: the wild-type cells as well as MCtrl with a heterozygous mutation. The later was chosen, as the expression level of miR-200c was not significantly changed, and the clone went through the same procedures as the miR-200c KOs. Therefore, it served as an appropriate control, to rule out expression changes based solely on selection and the introduction of TALENs proteins.

The PCA underlines the similarity of MCtrl to the wild type, prompting us to regard both as control groups. Moreover, a clustering analysis shows a close correlation between the replicas, as well as a tendency towards grouping the KO clones close together. This indicates that the knock-out of miR-200c does not lead to dramatic changes in the proteome, but to a surgical change in key elements and pathways, which are important for tumorigenesis.

For a comprehensive overview of changed expression patterns, we utilized two independent bioinformatic methods. While the DAVID analysis is based on a p-value pre-filtered set of proteins, analyzing gene ontology annotations on a broad level, the GSEA-Tool generates results due to a list-walk enrichment scoring analysis. Both analyses showed similar results, while used for a different purpose. One aim was to investigate pathways that are attributed to miR-200c expression and are involved in previously described physiological processes in cancer, like change in metabolic processes, EMT^43,44^ and resistance to chemotherapeutics^19^. The DAVID Analysis enabled a global understanding of process-changes attributed to a small set of differentially regulated proteins, revealing changes in metabolism and cellular organization in general. With the GSEA, we were able to analyze certain crucial pathways in cancer in detail, revealing changes in cancer progression and metastasis.

This is shown by enrichments in pathways increasing metabolic activity, loss of cell cycle regulation and actin cytoskeleton as well as cell-cell contacts. These findings, based on changes of protein expression were successfully correlated to phenotypes of the cells after the KO. After eradication of miR-200c expression, the cells showed increased migration, which could be attributed to changes in focal adhesion and cellular interaction. Also, increased NADP(H) turnover, as measurement of proliferation and metabolic activity is observed in the KO clones, which may also contribute to the increased resistance to doxorubicine treatment. The latter can also be caused by an increase of detoxification and the evasion of apoptosis.

While we do see changes in pathways and targets involved in cell motility and morphology as well as a changed phenotype towards more migratory cells, common EMT markers like vimentin were not found and E-cadherin expression was unchanged.

Epithelial MCF7 cells express low levels of ZEB1/2, as was confirmed previously^45^. Our data suggests that the miR-200c KO as such does not lead to an activation of ZEB1/2 and eventually to a decrease of E-cadherin. This may be due to the poised chromatin structures^46^ and not due to a persistent down-regulation via miR-200c. Our data suggests that miR-200c has additional effects on the cytoskeletal organization besides the ZEB1/2 axis, as was also proposed before^47^.

In more detail, the analysis of 675 proteins showed significant differential expression in 21 proteins in total, nine of those to a high extend in all three biological replicas. None of the obtained targets shown in Tables 1–3 were published to be regulated by miR-200c before. Comparing a list of confirmed miR-200c targets^12^ to our whole proteomics dataset, we found only 1 of 37 to be present, i.e. PRDX2. This protein displayed no significant differential expression in our analysis. The lack of prominent miR-200c targets in our tables may be based on different cell line models, as well as different analytical and experimental approaches used in the studies. Our proteomic approach as method does not allow gathering information of the whole proteome. Still, in this case the analysis of protein expression compared to a transcriptomic method may be beneficial, due to mainly translational changes which are expected after a miRNA KO. Nevertheless, on the basis of our data, it cannot be excluded that certain miR-200 family members may facilitate the regulation of certain proteins, without changing their own expression. Even the low expression of miRNAs may be enough to regulate translation, especially for low abundant proteins which often cannot be detected appropriately in proteomics approaches.

The regulatory mechanisms of miR200c seems to be different in our model cell line MCF7. MCF7 cells show high expression of miR-200c and as miRNA-inhibition is not very common this cell line model is not frequently used in miR-200c research. Consequently, most published miR-200c targets were discovered in other cellular systems. Additionally, the KO of an inhibitor leads to different results than the addition/overexpression of it, which was performed in the majority of the published studies. In a KO only physiologically direct targets and corresponding downstream effects become obvious while other inhibitory mechanisms (e.g. DNA methylation) are not affected by the KO and thus these potential miR-targets display no altered expression.

Moreover, we were analyzing a KO which is a longterm effect and might display different changes than those observed in transient overexpression or inhibition models. Transient experiments additionally may lack compensatory mechanisms.

While transient inhibition of miR-200c has revealed several functions in breast cancer, the long-term disruption of the gene may be more similar to the setting in a tumor. Of note, it was shown that miR-200c expression can be lost due to locus methylation, leading to more aggressive breast cancer phenotypes^48^. With our approach we were able to discovered novel targets which are truly governed by miR-200c in MCF7 cells and thus might play crucial roles also in the normal cellular settings.

Based on the information from the GO-Database, these targets were allocated to their main biological function: Most of the proteins play a role in cellular processes involving the cytoskeleton, metabolism and detoxification. This supports previous studies of miR-200c’s function in EMT, proliferation and chemoresistance^19,20,43,44^, while additionally revealing yet unknown miR-200c downstream proteins.

Our findings were affirmed by validation of changes on mRNA level by RT-qPCR on a set of six novel miR-200c targets (namely FLNA, FLNB, AGR2, SCIN, GSTM3 and ALHD7A1), originating from different data-mining methods and pathways.

Filamins A and B (FLNA, FLNB) are members of the nonmuscle actin-binding protein family. While both proteins in our experiments are up-regulated, most of the data in breast cancer is focused on filamin A. As both filamins are ubiquitously expressed and show high structural similarity, the two may act synergistically^49^ and underlie similar regulation. Filamin A was correlated to breast cancer development and progression in an *ex vivo* analysis^50^. Further, filamins can cause cell migration and invasion, by mediation of HGF/c-MET signaling as shown in hepatocytes^51^, as well as via the interplay with Cyclin D in highly metastatic human MDA-MB-231 cells^52^, which lack the expression of miR-200c^19^. Notably, also in a set of miR-200c low triple negative breast cancers (including MDA-MB-231) it was reported that filamin A^53^ knock-down leads to increased chemosensitivity to docetaxel.

Different proteins may contribute to enrichments in metabolic and cellular processes, like the Anterior Gradient Protein 2 Homolog (AGR2). AGR2 has been shown to play a critical role in numerous cancers and other diseases^54^, but especially in breast cancer, a high AGR2 expression shows negative effects on survival of tamoxifen treated patients^55^. After overexpression *in vitro*, increased proliferation and drug resistance to cisplatin was shown in the A375 cell line^56^ and even an apoptotic bystander effect of cancer cells on normal cells was shown^57^. These findings suggest an influence of AGR2 on drug resistance and breast cancer progression and as the miR-200c knock-out significantly increases its expression, miR-200c may be an important regulatory system for AGR2 expression. GSTM3, glutathione S-transferase Mu3 is a member of the glutathione transferase superfamily, which are known to play an important role in different processes of detoxification, likely also of chemotherapeutic drugs^58^. Recent publications show that inhibiting glutathione transferases may overcome resistance to platin-based DNA damaging drugs^59^.

Furthermore, Adseverin, the Calcium-Dependent Actin Severing and Capping Protein (SCIN), has been shown to have effects on different cancers. While no observations in breast cancer were reported, previous data show that a silencing of SCIN leads to a decrease in proliferation of A549 and H1299 lung carcinoma cells^60^. SCIN was also described as a driver in metastasis and outcome marker in patients with gastric cancer^61^, as well as its role in mediation of cisplatin resistance in bladder cancer cells^62^. All these findings correlate with effects observed in loss-of-miR-200c scenarios, which according to our data leads to an increase in SCIN.

Aldehyde dehydrogenases are a family of proteins oxidating aldehydes to carboxylic acids in NADP(H) dependent manner. Due to xenobiotics, reactive oxygen species (ROS) accumulate, finally leading to oxidative stress. Brocker *et al*. suggest ALDH7A1 may play an important role in the defense of the cell against oxidative stress and its cytotoxicity^63^. As the cytotoxic effect of doxorubicin and similar drugs is in parts accounted to reactive oxygen species (ROS) and oxidative stress^64,65^, the loss of miR-200c may cause the increase of ALHD7A1, leading to an increase in resistance to these therapeutics.

In this study, we combined a miRNA knock-out with a proteome analysis to investigate long-term effects, analogue to the loss of miR-200c during tumor progression in patients. Thereby, we were able to confirm known mechanisms of miR-200c, as shown by enrichment and pathway analysis. Moreover, we unraveled a set of novel target candidates involved in those mechanisms and were able to confirm the predicted effects by biological assays. Our data further emphasizes the role of miR-200c in tumorigenesis and underscores its potential as biomarker as well as putative therapeutic agent for miRNA-based therapies.

## Materials and Methods

### Reagents

Puromycin dihydrochloride and Doxorubicine hydrochloride were obtained from Sigma-Aldrich (cat. P8833, D1515).

### Cell culture

MCF7 cells stably expressing eGFP were generated in our lab. The parental cells were acquired from Cell Line Service (Eppelheim, Germany), grown at 37 °C and 5% CO_2_ in high glucose DMEM (Sigma) supplemented with 10% fetal calf serum (FCS/Gibco), as well as the miR-200c KO clones M1, M2, M3 and MCtrl. All cells were routinely tested and confirmed as mycoplasm free.

### miR-200c knock-out

Analysis for putative CRISPR-Targets was performed via the CRISPR-Design Tool from Feng Zhang’s lab (http://crispr.mit.edu, last target review: 18th of January, 2017)^66^.

The TALENs pair was acquired from the TALENs Library of the Seoul National University (http://cge.ibs.re.kr/html/cge_en/)^32^, the binding sequences for left and right TALENs are: CTAATACTGCCGGGTAATGA, TCCCTGTGTCAGCAACATCCA – respectively, the target sequence is TGGAGGCCCCTG. In order to develop a stable miR-200c KO in MCF7 cells, 600,000 cells per well were seeded in a 6 well plate and transfected on the following day with 3 µg DNA (equimolar ratio of two TALENs and a reporter plasmid containing a puromycin resistance cassette and red fluorescence protein (RFP)) using K2 Transfection System (Biontex) according to the manufacturers protocol. Two days post transfection, the cells were selected with 1 µg/ml of puromycin for two weeks, followed by single cell dilution to obtain monoclonal cultures.

DNA was extracted using Phenol-Chloroform (both Sigma), and analyzed by the T7-Surveyor assay (NEB). In mutation-positive clones, a sequencing of the miR-200c gene locus was performed. Three homozygous miR-200c KO clones were acquired, called M1, M2 and M3. The reporter-plasmid (SBI cat. MIR-KO-200cHR-1), comprises a puromycin and RFP reporter.

### Sequencing

DNA was extracted from MCF7 miR-200c KO cells using the standard protocol (phenol-chloroform). Approximately 500 ng of DNA were used to amplify the miR-200c gene using the following primers: Forward *CTCGAGGCTCACCAGGAAGTGTCCCC* Reverse *ACGCGTCCTTGTGCAACGCTCTCAGC*. The PCR product was purified by a PCR purification Kit (Qiagen Cat. 28104) and finally 50–100 ng of purified PCR product was sequenced (GATC Biotech AG).

### miRNA quantitative RT-PCR

Approximately 600,000 cells of each clone were harvested and total RNA isolated from cells using miRCURY RNA Isolation Kit (Exiqon). cDNA synthesis was carried out by a microRNA specific reverse transcription and detection with the qScript microRNA cDNA Synthesis Kit and PerfeCta SYBR Green SuperMix (Quanta Biosciences) with RT-PCR detection on a LightCycler 480 (Roche). The expression of miR-200 family members (miR-141, miR-200a, miR-200b, miR-200c) was normalized to miR-191^67^, using the 2^−∆CT^ or 2^−∆∆CT^ method. The following list contains the primers used for analysis of miRNAs:

miR200c: *GCGTAATACTGCCGGGTAAT*; miR-191: GCGCAACGGAATCCCAAAAG; miR-141: GCGTAACACTGTCTGGTAAAGA; miR-200a: GAGTAACACTGTCTGGTAACGA; miR-200b: GCGTAATACTGCCTGGTAATGA; miR-429: GAGTAATACTGTCTGGTAAAACC.

### Sample preparation for proteomic analysis

Protein was extracted from approximately 6 × 10^6^ cells using lysis buffer containing 8 M urea and 400 mM NH_4_HCO_3_. Briefly, cells were washed three times with cold PBS, treated with lysis buffer and harvested using cell scrapper. Lysates were concentrated with QIA-shredder mini spin column (Qiagen, Germany) following manufacturer’s instruction. Protein quantifications were performed using BCA Protein Assay Kit (Thermo Fisher Scientific). 20 µg of protein were prepared for disulfide bond reduction by adding 45 mM of dithioerythritol (DTE), and incubated for 30 min at room temperature. Alkylation of cysteines was performed by adding 0.1 M iodocetamide, followed by 30 min incubation at room temperature in the dark. Water was added to a concentration of 1 M urea. 400 ng sequencing grade modified porcine trypsin (Promega, Madison, WI, USA) was added for overnight incubation at 37 °C. Afterwards, samples were purified using C18 spin columns (Pierce, Thermo Scientific, IL, USA) complying manufacturer’s instruction. Resulting supernatants were combined and freeze-drying was performed. Peptide samples were stored at −20 °C prior to LC-MS/MS.

### Proteomic LC-MS/MS analysis

Samples were diluted in 0.1% formic acid. Nano-LC separation was done with a nano-liquid chromatography system (EASY-nLC 1000, Thermo Scientific, USA). 2.5 µg of peptide samples were loaded onto a trap column (PepMap100 C18, 75 µm × 2 cm, 3 µm particles, Thermo Scientific) and separated at a flow rate of 200 nl/min by an analytical reversed phase column (PepMap RSLC C18, 75 µm × 50 cm, 2 µm particles, Thermo Scientific) using a 260 min gradient from 5% B to 25% B (solvent A: 0.1% formic acid; solvent B: CH3CN/0.1% formic acid) followed by a 60 min gradient from 25% to 50% B. Tandem mass spectrometry was performed with an Orbitrap XL mass spectrometer (Thermo Scientific, USA). MS and MS/MS spectra were acquired using cycles of one MS scan (mass range m/z 300–2000) and five subsequent data dependent CID MS/MS scans (dynamic exclusion activated; collision energy: 35%).

### Analysis of proteomic data and bioinformatics processing

All data were processed with MaxQuant and analyzed in Perseus (version 1.5.3.2)^68–70^ at an FDR of 1% for the peptide and protein level. In Perseus, following operations were performed: Transformation (log2) and removal of possible contaminants and false positive identifications from the reversed database. For relative quantification, only those proteins were considered that showed valid LFQ-values in all three replicas in all samples. No imputation was performed.

In addition, proteins were considered “ON” when at least 5 valid values were found in M1, M2 and M3, and no value in the control. Proteins were considered “OFF” when at least 3 valid values were found in MCtrl and MCF7 and not at all in the KO group.

For pathway analysis, the whole data set was re-analyzed: After transformation and removal of contaminants and false positives, data was filtered for proteins found at least 3 times in one of the groups KO or Ctrl. The whole dataset was analyzed by the Gene Set Enrichment tool (GSEA, version 3.0 beta2)^35^, following the authors’ instructions. For analysis with DAVID Bioinformatics 6.7^34^, proteins were chosen which showed p ≤ 0.05 in a two-tailed student’s t-test, comparing Ctrl to KO group.

### Analysis of miR-200c binding in genes of target proteins

For the analysis of a potential miR-200c binding in the found genes, the Targetscan 7.1 database^71^ was used.

### Analysis of transcription factors in promoter regions

For the analysis of the promoter region, each gene’s sequence was retrieved from the RefSeq-Database (https://www.ncbi.nlm.nih.gov/refseq/ as of April 2017) in order to identify the +1 position. Assuming the +1 position as starting site of transcription, 500 nucleotides upstream were defined as the proximal promoter. Then, for analysis of proximal promoters, PhysBinder^72^ software was used and the analysis was performed with the highest stringency. The resulting transcription factors were evaluated for miR-200c and family binding with Targetscan 7.1^71^.

### qPCR validation of mRNA Expression

RNA was extracted utilizing the Total RNA Kit, peqGOLD (VWR) as by manufacturer’s instructions. Synthesis of cDNA was performed utilizing the qScript cDNA synthesis kit (Quanta Bioscience) as by manufacturer’s protocol.

Analysis of expression was performed with the Lightcycler 480 (Roche) and the Universal Probe Library (Roche) with following probe and primer (forward/reverse) combinations:

AGR2, Probe 47, GGTGGGTGAGGAAATCCAG/GTAGGAGAGGGCCACAAGG

ALDH7A1, Probe 7, CACTCAGGTGGGAAAACAGG/AATGGCATTGTTTCCTCCAA

FLNA, Probe 32, TCGCTCTCAGGAACAGCA/TTAATTAAAGTCGCAGGCACCTA

FLNB, Probe 21, CGGACTTCGTGGTAGAATCC/TGAGAGGGGCCTTCAATG

GSTM3, Probe 85, CCAATGGCTGGATGTGAAAT/TCCAGGAGGTAGGGCAGAT

SCIN, Probe 19, TTTCAAAGGCGGTCTGAAAT/CAGGTCGTTCGTAAGAACATGA

### Measurements of metabolic activity

All clones were seeded triplicates in a concentration of 5000 cells/well in four identical 96-well plates. The cells were treated with 10 µl of 5 mg/ml MTT (Sigma Aldrich) at the timepoints 0 h (about 2 h after seeding) and 24 h, 48 h and 72 h later respectively. The plates were incubated for 2 h at 37 °C and stored at −80 °C over night. Afterwards100 µl DMSO (Sigma Aldrich) were added and incubated for 37 °C for 30 mins, while shaking. Measurements were performed with the Spark 10 M (TECAN).

### Live cell imaging and 2D Migration

Live Cell Imaging was performed using a Nikon Eclipse Ti Inverted Microscope (Nikon, Düsseldorf, Germany). Cells were kept under constant 37 °C, 5% CO2 and 80% humidity by the heating and incubation system from Ibidi (Martinsried, Germany). Imaging was performed with the 10× phase contrast objective. For the 2D migration experiments 8-well slides (Ibidi, Martinsried, Germany) were coated with 50 µg/ml fibronectin for 1 h, afterwards cells were seeded in a density of 25 × 10^3^/well, and were allowed to attach to the coated surface for 2 h. Cell Displacement was imaged every 10 min over 20 h in all settings. For analysis of movement, single cells were tracked manually using ImageJ Manual Tracking Plugin. Acquired trajectories in 2D were further analyzed for mean velocity using Ibidi Chemotaxis and migration tool, afterwards an outlier-analysis was performed by the Identify outliers tool of Prism GaphPad.

### Clonogenic assay

1000 cells were seeded in a 6-well plate (TPP, Switzerland), and grown for 7 days, fixed and stained with paraformaldehyde (PFA) containing crystal violet (Sigma). Survival colony were analysed by ImageJ ColonyArea

### Doxorubicine resistance

All clones were seeded in a concentration of 5000 cells per well in 96 well plates. 24 h after seeding, cells were treated with 1 µM Doxorubicine for 48 h (Sigma Aldrich, stock 10 mM in DMSO). Analysis of viability was performed via Celltiter-Glo assay (Promega) and normalized to DMSO control.

### Statistical analysis

Results are expressed as the mean ± SD of at least three biological replicas, if not stated otherwise. Software GraphPad Prism v6 and SigmaPlot 11 were utilized for the analysis of the data. For analysis of miR-200c expression (only one variable and more than two groups), the One Way Analysis of Variance test was used, followed by the two tailed Bonferroni’s multiple comparison test, with DF = 4. For analysis of all family members (two variables and more than two groups per variable), the Two Way Analysis of Variance test was used, followed by two tailed Bonferroni’s multiple comparison test with DF = 16. For analysis of family expression between early and late passage (three variables and more than two groups per variable) we used the Three Way Analysis of Variance test, followed by two tailed Bonferroni’s multiple comparison test, with DF = 12.

### Data availability

The data that support the findings of this study are available from the corresponding author upon reasonable request.

## Electronic supplementary material

Supplementary Information
