# Supplementary material for: A proteomic analysis of an in vitro knock-out of miR-200c
Source: Sci Rep. 2018 May 2;8:6927. doi: 10.1038/s41598-018-25240-y (PMC5931999; doi:10.1038/s41598-018-25240-y)
Supplement: Supplementary file 1 — Supplementary Information [file 41598_2018_25240_MOESM1_ESM.pdf]

A proteomic analysis of an in vitro knock-out of miR-200c

Bojan Ljepoja[1#¶], Jonathan García-Roman[1#¶], Ann-Katrin Sommer[1], Thomas Fröhlich[2], Georg J. Arnold[2], Ernst Wagner[1], Andreas Roidl[1]*

[1]*Pharmaceutical Biotechnology, Department of Pharmacy, Ludwig-Maximilians-Universität München, Munich, Germany*

[2]*Laboratory for Functional Genome Analysis (LAFUGA), Gene Center, Ludwig-Maximilians-Universität München, Munich, Germany*

* E-mail: andreas.roidl@cup.uni-muenchen.de

[#] These authors contributed equally to this work.

S1

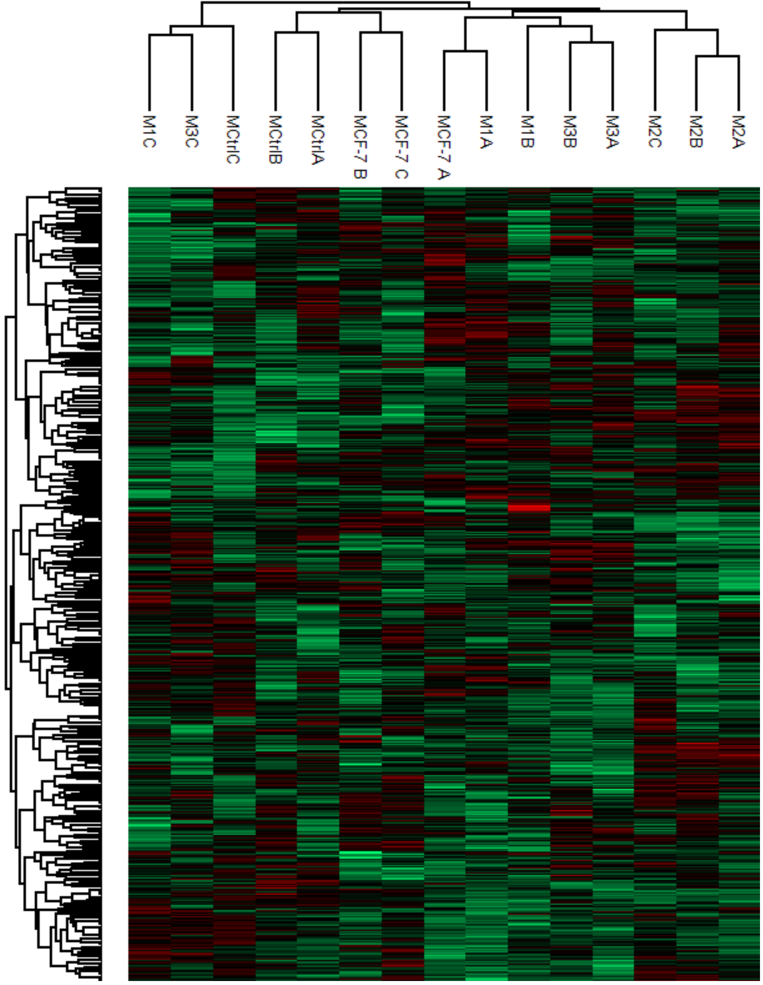

Figure S1 Clustering analysis of the measurements after vertical and horizontal z-score normalization

S2

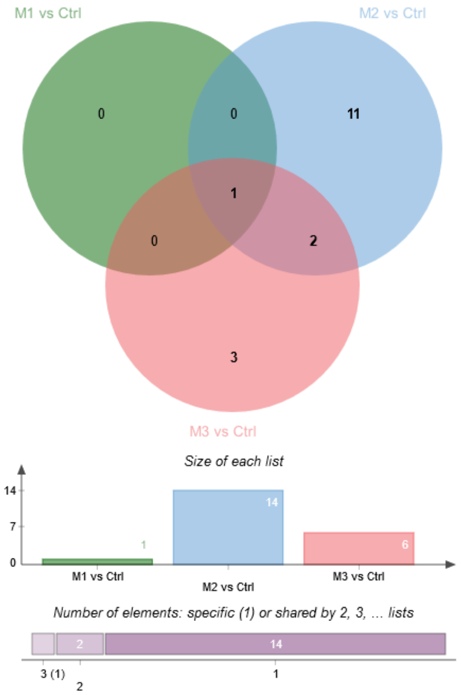

Figure S2 Venn-diagramm with results of the vulcano blot analysis of M1 or M2 or M3 vs (MCF7 and MCtrl) each N=675 with 250 randomizations, FDR 0.05 and S0 of 0.1 as shown in Table 2

S3

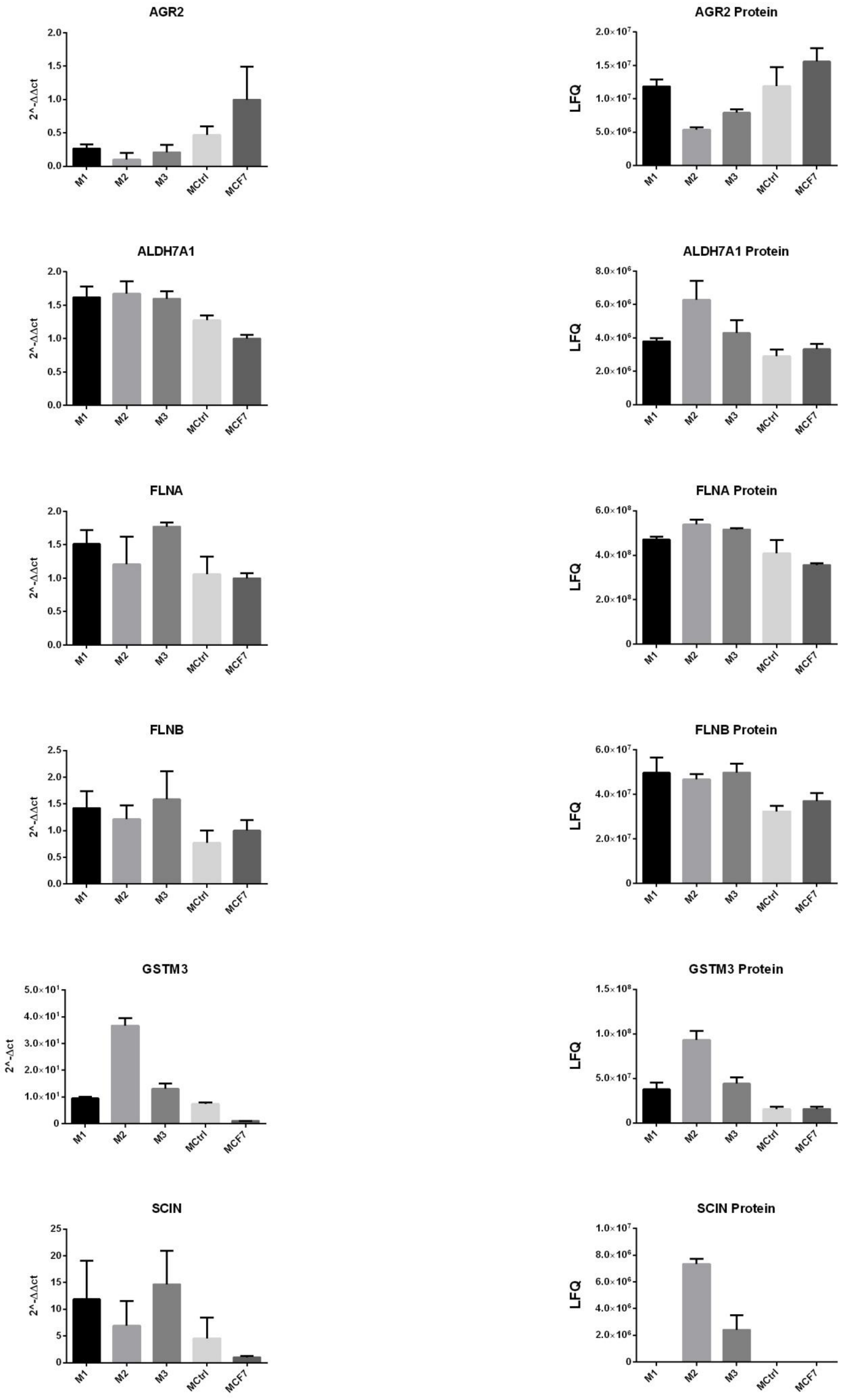

Figure S3 Detailed results of Figure 3F, with mRNA measurements compared to the protein expression data for each clone

S4

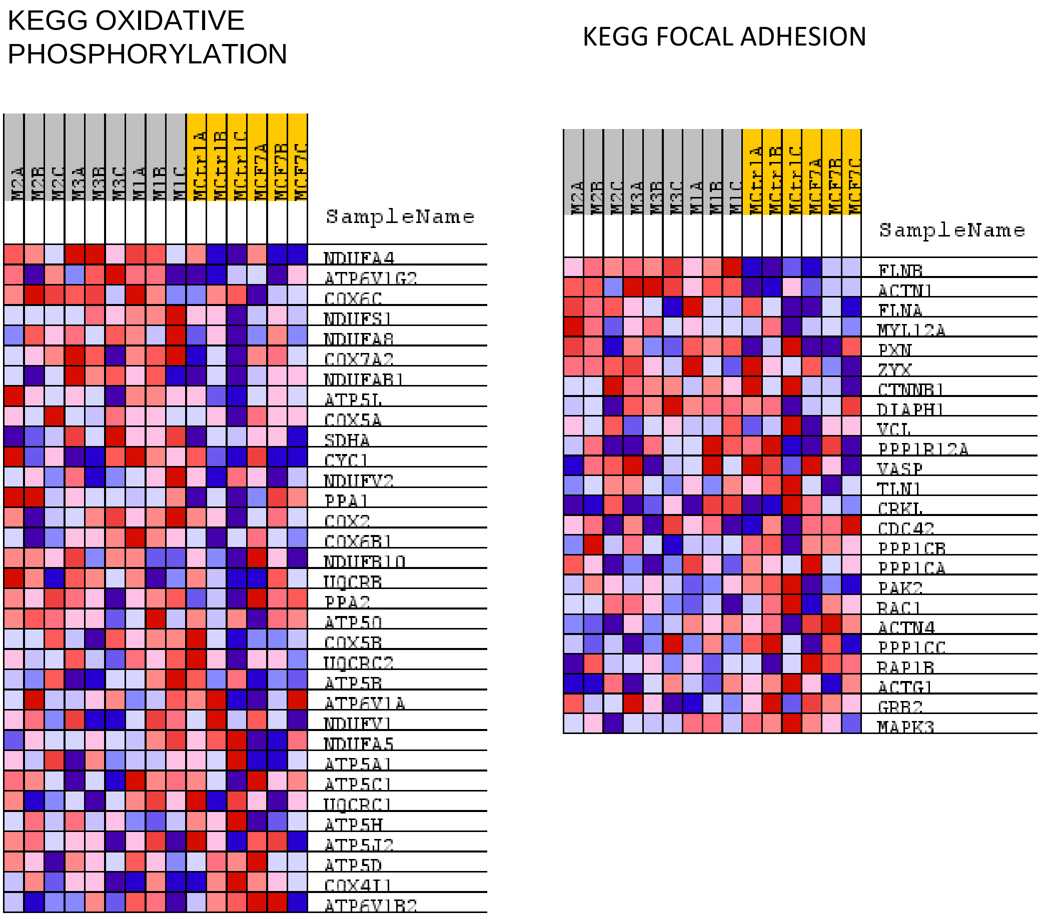

Figure S4 Heatmaps corresponding to the Enrichment blots in Figure 4C

S5

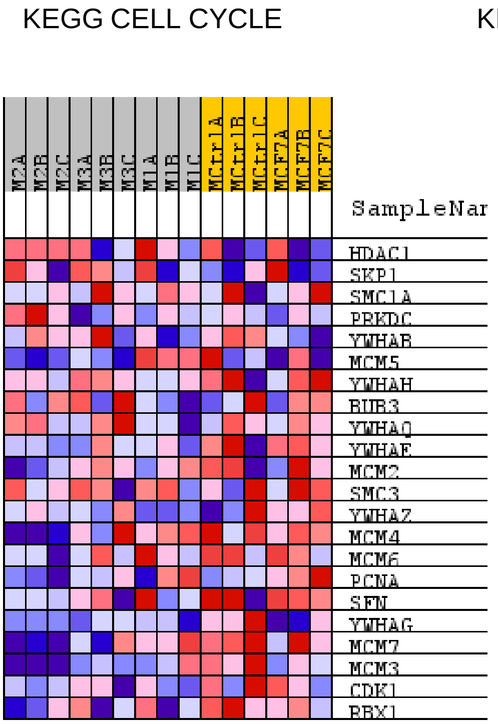

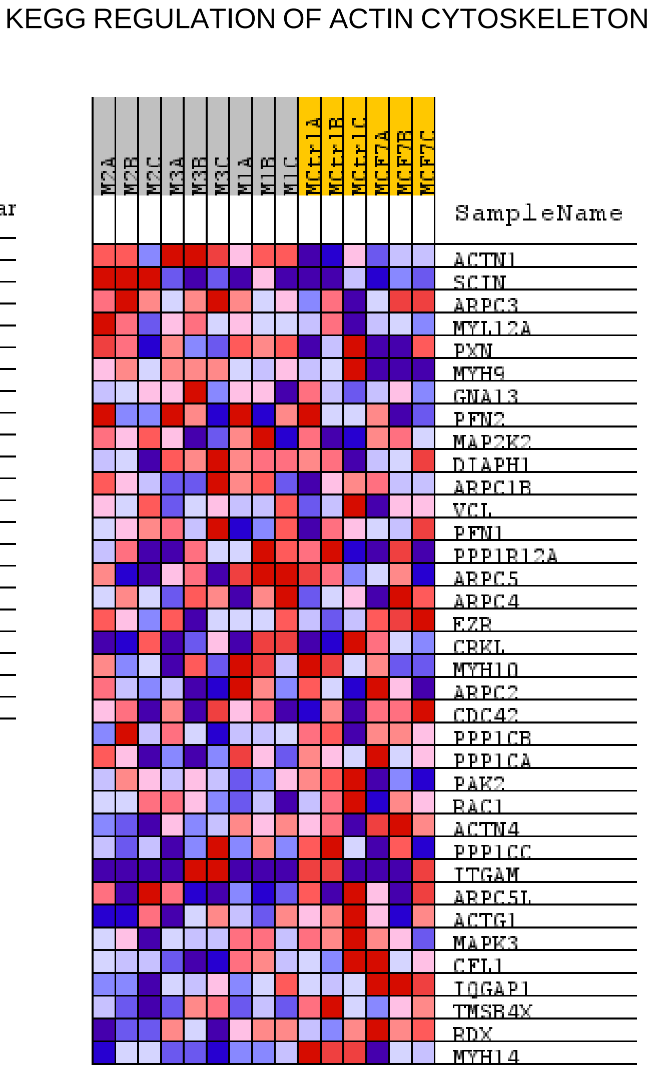

Figure S5 Heatmaps corresponding to the Enrichment blots in Figure 4D

S6

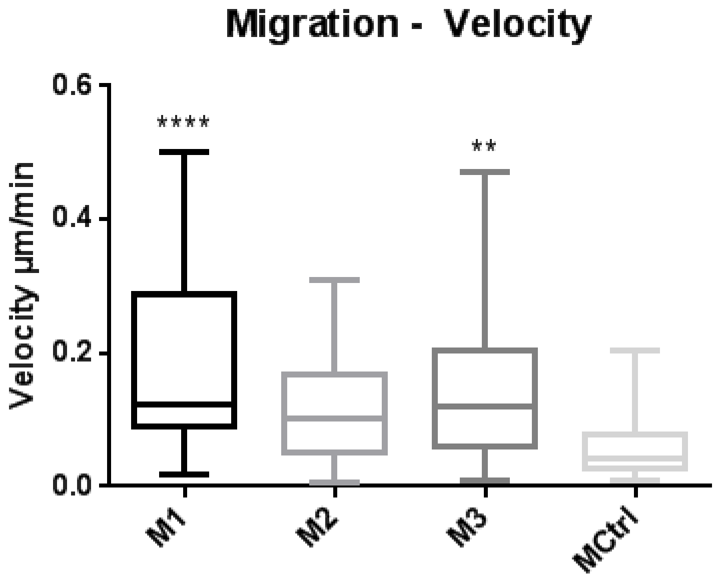

Figure S6 Evaluation of migration data in Figure 5B (N=30, *p≤0.05, one-way ANOVA with post-hoc Bonferroni's multiple comparison)

S7

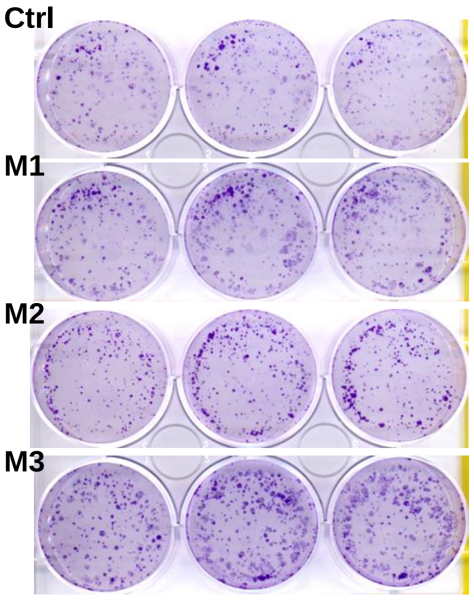

Figure S7 Clonogenic assay – imaging of colonies, as evaluated in the pooled analysis in figure 5d

S8

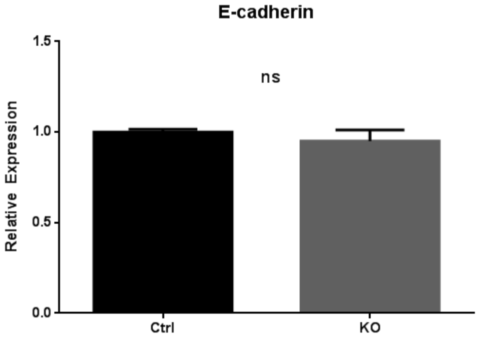

Figure S8 qPCR measurements of E-Cadherin mRNA levels in Ctrl vs KOs shows no significant difference

Table S1 15 / 32 gene sets are enriched in phenotype KO

NAME	SIZE	ES	NES	NOM p-val	FDR q-val	FWER p-val	RANK AT MAX	LEADING EDGE
KEGG_PARKINSONS_DISEASE	34	0.46907002	15.940.783	0.009861933	0.24380365	0.214	345	tags=50%, list=28%, signal=67%
KEGG_OXIDATIVE_PHOSPHORYLATION	33	0.48710477	15.471.032	0.018480493	0.17806831	0.293	345	tags=52%, list=28%, signal=70%
KEGG_ALZHEIMERS_DISEASE	35	0.420356	14.566.842	0.046	0.24040706	0.479	292	tags=40%, list=24%, signal=51%
KEGG_CITRATE_CYCLE_TCA_CYCLE	20	0.4615872	13.821.458	0.12048193	0.29699662	0.648	132	tags=30%, list=11%, signal=33%
KEGG_HUNTINGTONS_DISEASE	43	0.36943752	13.720.317	0.03508772	0.2523027	0.664	292	tags=42%, list=24%, signal=53%
KEGG_GLUTATHIONE_METABOLISM	15	0.44105875	13.544.401	0.08317215	0.23395455	0.703	78	tags=27%, list=6%, signal=28%
KEGG_MAPK_SIGNALING_PATHWAY	16	0.4151188	13.413.316	0.10453649	0.22068681	0.734	136	tags=25%, list=11%, signal=28%
KEGG_AMINOACYL_TRNA_BIOSYNTHESIS	20	0.44356683	13.299.485	0.15369262	0.20533033	0.744	263	tags=40%, list=21%, signal=50%
KEGG_CARDIAC_MUSCLE_CONTRACTION	17	0.46564567	12.798.785	0.18257262	0.23733874	0.82	292	tags=53%, list=24%, signal=68%
KEGG_ANTIGEN_PROCESSING_AND_PRESENTATION	15	0.42715377	12.191.079	0.24395162	0.285524	0.892	308	tags=47%, list=25%, signal=61%
KEGG_PYRUVATE_METABOLISM	15	0.49441242	11.757.169	0.28846154	0.31522772	0.919	122	tags=33%, list=10%, signal=37%
KEGG_GLYCOLYSIS_GLUCONEOGENESIS	23	0.39739954	11.214.875	0.33840305	0.35995352	0.95	200	tags=30%, list=16%, signal=36%
KEGG_FOCAL_ADHESION	24	0.30988422	10.625.255	0.36055776	0.41783723	0.972	277	tags=29%, list=22%, signal=37%
KEGG_LYSOSOME	17	0.24309203	0.69935	0.9089184	0.94843	1.0	162	tags=18%, list=13%, signal=20%
KEGG_LEUKOCYTE_TRANSENDOTHELIAL_MIGRATION	17	0.19984435	0.660395	0.934236	0.92140806	1.0	195	tags=18%, list=16%, signal=21%

Table S2 17 / 32 gene sets are upregulated in phenotype Ctrl

NAME	SIZE	ES	NES	NOM p-val	FDR q-val	FWER p-val	RANK AT MAX	LEADING EDGE
KEGG_CELL_CYCLE	22	-0.51119137	-16.219.078	0.035643563	0.19916053	0.172	348	tags=55%, list=28%, signal=75%
KEGG_RIBOSOME	68	-0.31785846	-14.187.368	0.016746411	0.49701187	0.529	392	tags=41%, list=32%, signal=57%
KEGG_ENDOCYTOSIS	20	-0.40655762	-13.007.351	0.12331407	0.70801485	0.78	193	tags=30%, list=16%, signal=35%
KEGG_PURINE_METABOLISM	23	-0.36597934	-11.775.029	0.24390244	0.9449636	0.925	14	tags=13%, list=1%, signal=13%
KEGG_PROTEASOME	34	-0.30099234	-11.316.409	0.29045644	0.9364207	0.95	457	tags=53%, list=37%, signal=82%
KEGG_ADHERENS_JUNCTION	15	-0.35883263	-11.289.837	0.28879312	0.78668696	0.95	338	tags=53%, list=27%, signal=73%
KEGG_TIGHT_JUNCTION	20	-0.3472254	-10.904.311	0.3391473	0.7903667	0.964	294	tags=40%, list=24%, signal=52%
KEGG_OOCYTE_MEIOSIS	19	-0.3068673	-0.99786586	0.49278352	0.9669546	0.988	440	tags=58%, list=36%, signal=89%
KEGG_UBIQUITIN_MEDIATED_PROTEOLYSIS	16	-0.319742	-0.979242	0.4989059	0.909404	0.991	251	tags=31%, list=20%, signal=39%
KEGG_INSULIN_SIGNALING_PATHWAY	17	-0.29883376	-0.95862424	0.51827955	0.8756116	0.994	256	tags=35%, list=21%, signal=44%
KEGG_REGULATION_OF_ACTIN_CYTOSKELETON	36	-0.24238425	-0.9287425	0.6079295	0.8686062	0.998	275	tags=31%, list=22%, signal=38%
KEGG_PATHOGENIC_ESCHERICHIA_COLI_INFECTION	22	-0.28710213	-0.9233835	0.595092	0.8081302	0.998	514	tags=55%, list=42%, signal=92%
KEGG_NEUROTROPHIN_SIGNALING_PATHWAY	17	-0.29578927	-0.90542006	0.5875831	0.7824528	0.999	440	tags=59%, list=36%, signal=90%
KEGG_SPLICEOSOME	58	-0.22438549	-0.8703807	0.6956522	0.7967216	0.999	364	tags=38%, list=29%, signal=51%
KEGG_PATHWAYS_IN_CANCER	27	-0.22919808	-0.7894147	0.8729839	0.8798361	0.999	338	tags=41%, list=27%, signal=55%
KEGG_FC_GAMMA_R_MEDIATED_PHAGOCYTOSIS	16	-0.24920684	-0.7617598	0.872	0.8646141	1.0	218	tags=25%, list=18%, signal=30%
KEGG_SYSTEMIC_LUPUS_ERYTHEMATOSUS	16	-0.28722718	-0.7255098	0.8017058	0.8583328	1.0	362	tags=44%, list=29%, signal=61%

Table S3 – Overview of predicted transcription-factor binding sites

Gene	Quality* of predicted miR-200c binding-site
**ALDH7A1**	7mer-m8
**CA2**	7mer-A1
**GSTM3**	7mer-m8
**KYNU**	8mer / 7mer-m8 / 7mer-A1
**SCIN**	8mer / 7mer-A1

*** as described on https://www.targetscan.org**

	TF with predicted miR-200c binding site																																												
TF ➔ / Gene ↓	KLF4	ZFX	EGR1	NRF1	ELK4	ETS1	GABPA	ZEB1	FOSL2	JUN	SP1	STAT1	TFAP2A	GATA2	MYC	Tfcp2l1	Esrrb	RELA	Sp2	XBP1	CTCFL	Sp4	ELF1	TFAP2C	RUNX3	MEF2C	CTCF	GTF3C2	RAD21	BRCA1	GATA1	MAFF	USF2	YY1	E2F1	ELK1	ESRRA	FOS	IRF3	JUNB	Mycn	REST	TAF1	TAF7	BHLBE40
ASMTL	x	x		x												x	x		x	x	x									x															
CTTN	x	x	x		x	x	x		x		x		x		x	x	x	x			x	x	x	x	x			x	x	x	x					x		x		x					x
FLNA	x	x	x						x	x	x					x	x	x	x	x	x	x		x	x		x	x									x	x	x	x					
FLNB	x	x	x	x									x			x	x	x	x	x	x	x	x	x		x	x			x															
GOT2	x	x														x	x	x											x		x														
HSP90AA1	x			x												x	x		x	x			x	x										x								x	x	x	
PDXK	x	x	x	x		x		x								x	x		x	x		x		x	x			x	x																
PPP1CC	x	x	x		x	x								x		x	x	x	x	x	x	x	x																						
PYCARD	x	x										x				x	x	x		x	x			x					x																
RAB14	x			x												x	x	x								x						x													
RMDN1	x	x	x												x	x	x	x	x	x	x	x	x		x		x					x	x								x				x
SLC3A2	x			x												x	x	x	x			x									x			x											
SPTAN1	x		x		x		x									x	x		x	x	x	x	x																						
TPM1	x		x													x	x	x			x				x	x																			
UGDH	x	x	x		x		x	x								x	x	x	x	x	x	x	x										x			x									
TPI1	x																																												
AGR2																x	x	x							x	x																			
SH3BGRL																x		x																											
PHGDH	NA																																												
MCM4	NA																																												
WARS	NA																																												

TF: Transcription Factor, NA: Algorithm was not providing any binding site or the intergenic region was too short for valid prediction
